# Machine learning-based prediction of insufficient contrast enhancement in coronary computed tomography angiography

**DOI:** 10.1007/s00330-022-08901-5

**Published:** 2022-06-16

**Authors:** R. R. Lopes, T. P. W. van den Boogert, N. H. J. Lobe, T. A. Verwest, J. P. S. Henriques, H. A. Marquering, R. N. Planken

**Affiliations:** 1 Department of Biomedical Engineering and Physics, Amsterdam UMC, University of Amsterdam, Amsterdam, The Netherlands; 2 Department of Radiology and Nuclear Medicine, Amsterdam UMC, University of Amsterdam, 1105 AZ Amsterdam, The Netherlands; 3 Department of Clinical and Experimental Cardiology, Amsterdam UMC, University of Amsterdam, Amsterdam Cardiovascular Sciences, Amsterdam, The Netherlands

**Keywords:** Computed tomography angiography, Computed tomography coronary angiography, Contrast delivery protocol, Contrast enhancement, Machine learning

## Abstract

**Objectives:**

Patient-tailored contrast delivery protocols strongly reduce the total iodine load and in general improve image quality in CT coronary angiography (CTCA). We aim to use machine learning to predict cases with insufficient contrast enhancement and to identify parameters with the highest predictive value.

**Methods:**

Machine learning models were developed using data from 1,447 CTs. We included patient features, imaging settings, and test bolus features. The models were trained to predict CTCA images with a mean attenuation value in the ascending aorta below 400 HU. The accuracy was assessed by the area under the receiver operating characteristic (AUROC) and precision-recall curves (AUPRC). Shapley Additive exPlanations was used to assess the impact of features on the prediction of insufficient contrast enhancement.

**Results:**

A total of 399 out of 1,447 scans revealed attenuation values in the ascending aorta below 400 HU. The best model trained using only patient features and CT settings achieved an AUROC of 0.78 (95% CI: 0.73–0.83) and AUPRC of 0.65 (95% CI: 0.58–0.71). With the inclusion of the test bolus features, it achieved an AUROC of 0.84 (95% CI: 0.81–0.87), an AUPRC of 0.71 (95% CI: 0.66–0.76), and a sensitivity of 0.66 and specificity of 0.88. The test bolus’ peak height was the feature that impacted low attenuation prediction most.

**Conclusion:**

Prediction of insufficient contrast enhancement in CT coronary angiography scans can be achieved using machine learning models. Our experiments suggest that test bolus features are strongly predictive of low attenuation values and can be used to further improve patient-specific contrast delivery protocols.

**Key Points:**

*• Prediction of insufficient contrast enhancement in CT coronary angiography scans can be achieved using machine learning models.*

*• The peak height of the test bolus curve is the most impacting feature for the best performing model.*

**Supplementary Information:**

The online version contains supplementary material available at 10.1007/s00330-022-08901-5.

## Introduction

Computed tomographic coronary angiography (CTCA) is a non-invasive imaging technique used for the anatomical assessment of coronary artery disease [1–6]. Iodine containing contrast material (CM) is used to enhance luminal attenuation to enable assessment of the coronary artery lumen, vessel wall, and the surrounding structures [7]. Adjustments in CM delivery protocols change the attenuation coefficient of the blood pool. A commonly used strategy to adjust CM delivery is to regulate the iodine delivery rate (IDR = amount of iodine injected per second [g I/s] = concentration of CM × flow rate in ml/s) [7]. Besides CM delivery, coronary lumen attenuation also depends on patient features like body weight and length as well as CT scanner settings and the tube voltage (kV) in particular [7]. Other parameters, such as the peak height and time to peak of a test bolus, are also associated with attenuations but are commonly not considered in current CM protocols [8, 9]. A better understanding of the interrelation between these parameters and luminal attenuation is valuable for further improvements in patient-specific contrast delivery protocols. Reducing the iodine load is important to lower the risk for renal function impairment, reduce environmental pollution, and lower overall costs. However, inappropriate correction in contrast administration may result in insufficient coronary lumen attenuation and this is not tolerable.

For accurate assessment of coronary artery disease on CTCA, intra-arterial attenuation values higher than 350 HU are recommended [10–15]. In previous studies, the introduction of patient-tailored CM protocols, adjusting the IDR for body weight and kV, resulted in more constant coronary artery attenuation values and a favorable reduction in total iodine load [8, 10–12]. However, in some cases, CM delivery resulted in low coronary attenuation values, thereby jeopardizing the diagnostic value of CTCA [8].

We hypothesized that machine learning (ML) can help to predict cases with insufficient contrast attenuation in CTCA. This will allow for CM delivery and CT scanner settings to enhance coronary attenuation and improve diagnostic value. Additionally, we investigated the added value of using the test bolus features for the prediction. To this end, we also analyzed the impact of the features on predicting insufficient attenuation.

## Materials and methods

### Study design and population

This retrospective study was performed following the principles of the Declaration of Helsinki and the local Institutional Review Board approved this study. The Ethics Committee approved this research with a waiver. All consecutive patients above 18 years old who underwent CTCA between September 2017 and September 2020 were included in the study. CT scans were excluded if the acquisition protocol deviated from the standard CTCA protocol (e.g., TAVI or cardiac function) or if the test bolus enhancement curves were not stored in the hospital’s picture archiving and communication system.

### CTCA acquisition protocol

The imaging protocol has been described before [8]. In summary, all images were obtained using a third-generation dual-source 192 detector row CT scanner (Somatom Force, Siemens Healthcare). Sublingual nitro-glycerine spray was administered before the CTCA acquisition and beta-blockers were administered on indication (heart rate > 65 per min). The time between the start of contrast medium injection and the time to peak contrast enhancement in the ascending aorta was determined using a test bolus injection with a fixed contrast bolus of 10 ml undiluted contrast medium (Ultravist 300: iopromide 300 mg I/ml, Bayer AG or Xenetix 350: iobitridol 350 mg I/ml, Guerbet OptiVantage DH) a fixed scan delay of 8 s and a fixed kV value of 100 kV. For timing the CTCA acquisition, the scan delay was determined by the time to peak and an additional 4 s for coronary artery filling. For the CTCA scans, automatic tube voltage selection (CARE kV, Siemens Healthcare) was applied in all patients with kV categories ranging from 70 to 120kV with increments of 10kV. All CTCA scans were visually evaluated by the attending CT technician. CT scanner acquisition parameters were: detector collimation 2 × 96 × 0.6 mm, slice acquisition 2 × 192 × 0.6 mm using a z-flying focal spot, gantry rotation time of 250 ms, temporal resolution of 66 ms, 70–120 kV tube voltage (CARE kV), and 180–600 μA tube current. High-pitch spiral scanning was performed in diastole in patients with a regular heart rate < 70/min. A prospective ECG-gated sequential scan (step and shoot) was performed in diastole for patients with irregular heart rate < 70/min or heart rates ranging between 70 and 80/min. For patients with irregular heart rates of > 80/min, a sequential scan was performed in systole. Padding in an adaptive prospective sequential scan mode for high and irregular heart rates was used to enable reconstruction of more cardiac phases. Images were reconstructed with a slice thickness of 0.6 mm and an increment of 0.4 mm using iterative reconstruction factor 2 (ADMIRE, Siemens Healthcare).

### Contrast delivery protocol

Iodinated contrast medium (300 or 350 mg I/ml) was administered via a dual-head contrast delivery injector (Guerbet OptiVantage DH) equipped with a high-pressure resistant extension tube and injected in the right antecubital vein. A test bolus of 10 ml contrast medium was injected at 6 ml/s or 6.5 ml/s, followed by a 40-ml saline chaser also injected at 6 or 6.5 ml/s. The bolus of (un)diluted contrast material for high-pitch spiral CTCA scans was 50 ml and the bolus for prospective sequential step-and-shoot scans was 65 ml. The larger contrast bolus volume in prospective sequential step-and-shoot scans was applied to compensate for the longer acquisition time. The contrast bolus was injected at an injection rate of 6 ml/s or 6.5ml/s. All contrast injections were followed by a saline chaser of 40 ml (6 or 6.5 ml/s). The IDR was adjusted for body weight and kV settings, as presented in a previous study [8]. The kV settings for CTCA acquisition, as selected by CARE kV, were used together with body weight to provide a patient-specific IDR (1–2.3 g I/s). To reach the required IDR, the CM was diluted with saline via the dual-head contrast delivery injector, of which one was filled with undiluted contrast material and the other with saline solution. The two fluids were blended in the high-pressure resistant extension tube, after which it was injected in the right antecubital vein.

### Data extraction

**Fig. 1:**
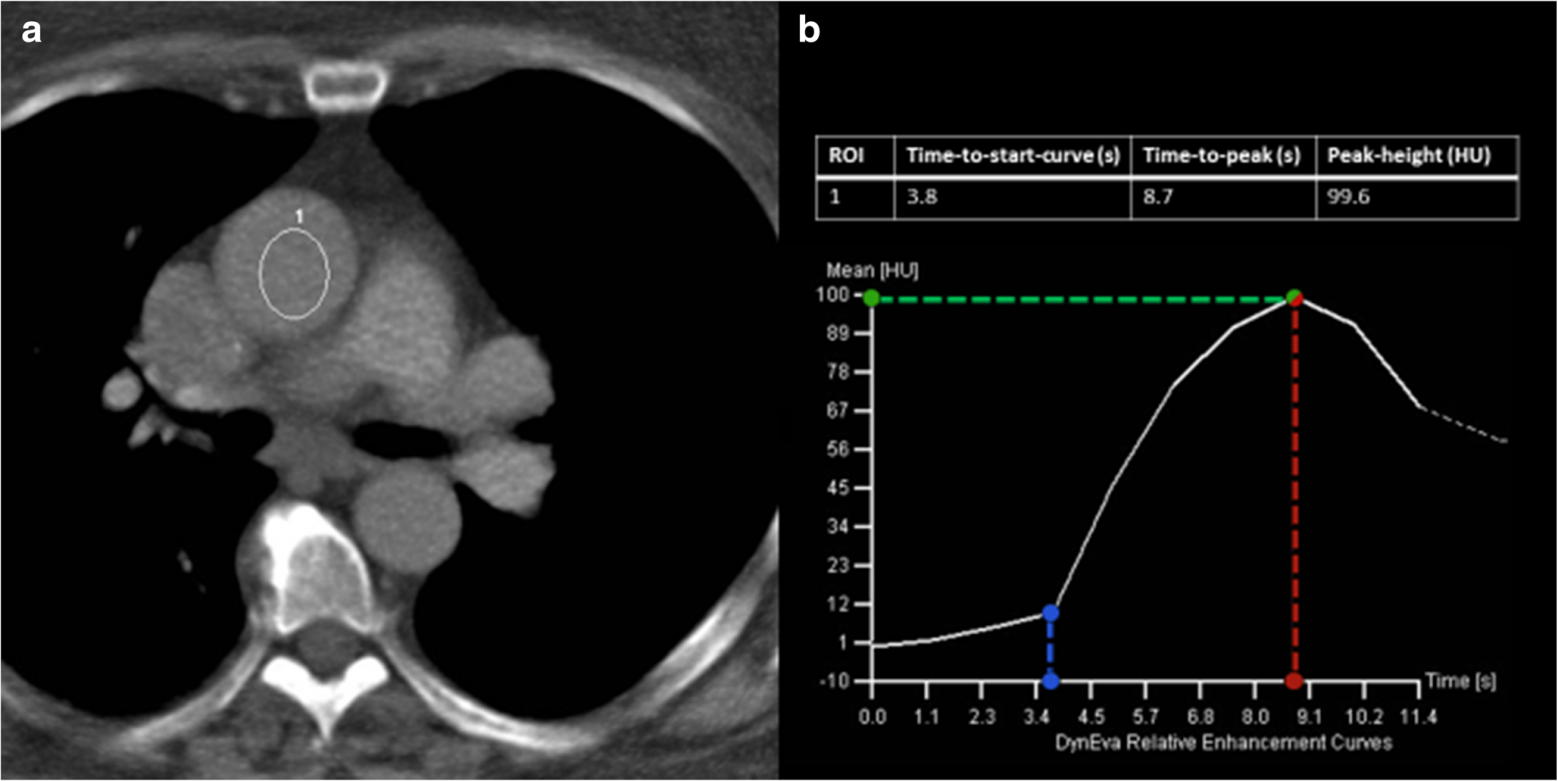
Dynamic bolus tracking of the test-bolus scan example. An ROI is used to measure the attenuation at the level of the ascending aorta below the level of the carina (**a**, 1). The curve (**b**) represents the measured values over time. Time-to-start-curve (blue dashed line) in seconds, time-to-peak (red dashed line) in seconds, and peak height (green dashed line) in HU where *t* = 0 corresponds to 8 s after contrast media injection

Data was retrieved automatically from DICOM headers and electronic patient records. Collected patient features included sex, age, average heart rate, body weight, and body height. Also, kV settings (tube voltage), iodine delivery rate (IDR), total iodine load, and contrast dose concentration were collected. Furthermore, we extracted test bolus features, such as the peak height of the test bolus attenuation curve (peak height in HU), the time to peak of the contrast curve (time-to-peak in seconds), and the time to the start of the contrast curve (the time-to-start-curve in seconds) from the bolus tracking curves (DynEva, Siemens Healthcare) as illustrated in Fig. 1. An association between the height of the test bolus and coronary attenuation has been reported in previous studies. Therefore, we considered the test bolus to contain important information and included this in the model [8, 9]. Regarding the time to start and time to peak, the default delay of 8 s was ignored in the analysis once the values used were obtained from the bolus tracking curves.

For the assessment of luminal attenuation, we used an in-house developed tool to automatically detect the ascending aorta. Correspondingly, a region of interest with a radius of approximately 70% of the aorta radius was fitted to calculate the average attenuation in the ascending aorta and exclude possible edges and calcifications in the vessel wall. In cases in which the tool did not detect the ascending aorta, the location was selected manually. The attenuation value in the ascending aorta was used as a proxy of the attenuation in the coronary arteries. It should be noted that the attenuation in the ascending aorta is slightly higher than that in the coronary arteries. Previous research has shown that there is a strong association between attenuation in the ascending aorta and coronary arteries with a mean decay of 25 HU expected from the ascending aorta to the proximal coronary arteries and of 50 HU to the distal coronary arteries [8]. Therefore, the cutoff value for adequate attenuation in the ascending aorta for this study was 400 HU.

### Model development

The models were trained to predict insufficient luminal attenuation in the ascending aorta. Insufficient attenuation was defined as an average attenuation lower than 400 HU within the region of interest. ML techniques were used to deal with both linear and nonlinear interactions between the included features. These techniques included the following: logistic regression (LR), random forest (RF), extreme gradient boosting, support vector machines, and neural networks. To assess the added value of extracting information from the test bolus, we performed two experiments: only patient features with CT settings and, additionally, also including the test bolus features. Both experiments followed the same methods and only differed in the features included.

We used stratified 10-fold cross-validation (CV) for the development and evaluation of the ML models. In some cases where CTs from the same patient were split into training and test set, the CTs were removed from the training set to avoid patient data leakage. The training set was also used to find the optimal hyper-parameters using a grid search with another 5-fold CV. For model selection after the hyper-parameter optimization, the models with the largest average area under precision (positive predictive value) and recall (sensitivity) curves were selected. The testing folds were not used during the training steps.

To deal with the missing values, we used MissForest, an iterative technique based on random forests [16] for imputation. The imputation model was created with the training data only to avoid data leakage. As a requirement for some of the ML techniques, the continuous features were standardized by removing their mean and scaling to unit variance.

Detailed information about the selected classifiers and hyper-parameters used for optimization is available in the Supplementary Material Tables I and II. The analysis was performed with Python (Python Software Foundation, version 3.6, www.python.org) using the scikit-learn [17] and XGBoost [18] packages.

### Model evaluation

The area under the receiver operating characteristic (AUROC) and precision-recall curves (AUPRC) were used to evaluate the models. As a 10-fold CV was applied, we computed the averages and 95% confidence interval (CI) for each model. The Wilcoxon signed-rank test was performed to assess whether the difference in AUROC and AUPRC between the prediction models with and without using the test bolus features are statistically significant (*p*-value < 0.05).

### Model interpretation

For the visualization of the importance of included features in the prediction analysis, the Shapley Additive exPlanations (SHAP) framework was used [19]. For each of the features, the feature importance (SHAP value) was calculated by making predictions excluding that feature. This value describes how it affects the prediction probability. The larger the SHAP value, the more it affects the prediction. Additionally, the values can be either positive, for low attenuations, or negative, for regular attenuations.

The SHAP values were computed for the entire population. In addition, for a better understanding of the effect of the features per tube voltage, we also computed the SHAP values per tube voltage group (70–120 kV).

## Results

**Fig. 2:**
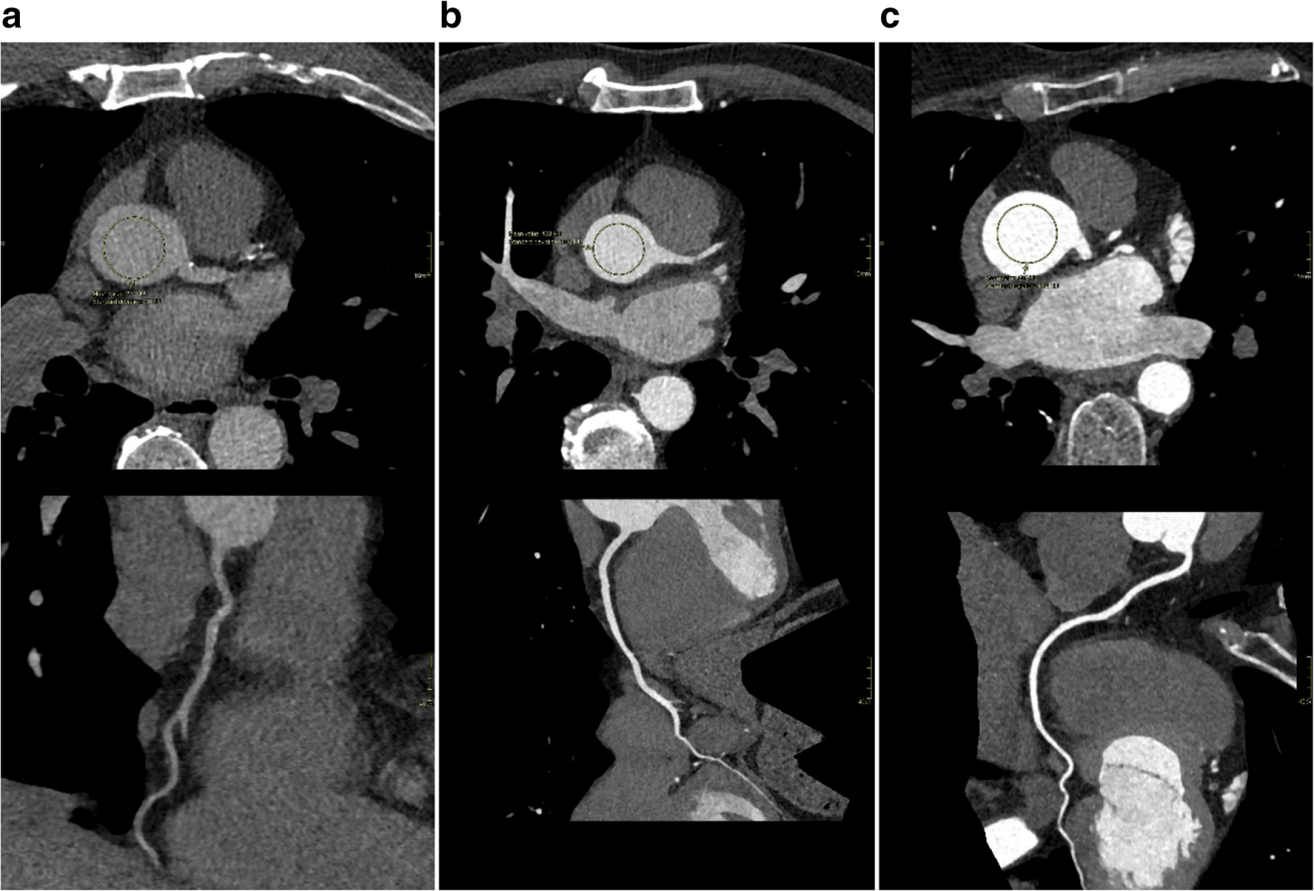
Examples of CTCA scan (axial slice through the ascending aorta above and curved multiplanar reconstruction of the right coronary artery below) of patients with low (**a**), accurate (**b**), and high (**c**) attenuation. The images are displayed with a window width of 800 and a window level of 200

A total of 1,447 scans from 1,364 patients were included in the analysis. Of these scans, 399 (27%) were considered to have insufficient attenuation. Figure 2 displays an example of CTCAs with insufficient (227 HU), accurate (433 HU), and high (595 HU) attenuation. The tool for automated ascending aorta detection failed in less than 1% of the cases. Baseline and descriptive features are shown in Table 1 and average attenuation values per tube voltage are shown in Table 2. The relationship between patient weight and mean attenuation per kV group is shown in Supplementary Material Figure I. Despite the already-applied correction for kV and body weight in our acquisition protocol, there was considerable variation between patients.

**Table 1:** Descriptive statistics of the study group, mean ± SD or *N* (%)

		Missing (*n*)	Low attenuation (*n* = 399)	Regular attenuation(*n* = 1048)
Age (years)		374	55.7 ± 11.8	52.8 ± 12.2
Sex (female)		374	494 (62%)	101 (37%)*
Height (cm)		568	171 ± 10	177 ± 10*
Weight (kg)		556	77 ± 14	86 ± 19*
Average heart rate (bpm)		0	61.9 ± 10.2	61.6 ± 11.2
Iodine delivery rate (g I/s)		0	1.5 ± 0.3	1.5 ± 0.3*
Tube voltage (kV)	**70**	0	357 (34%)	111 (28%)*
	**80**		426 (41%)	110 (28%)
	**90**		214 (20%)	75 (19%)
	**100**		29 (3%)	39 (10%)
	**110**		18 (2%)	23 (6%)
	**120**		4 (0%)	41 (10%)
Total iodine load (g)		0	16.0 ± 2.8	15 ± 2.7
Peak height-test bolus (HU)		0	127 ± 40	95 ± 35*
Time to peak-test bolus (s)		0	8.5 ± 2.7	9.6 ± 3.4*
Time to start-test bolus (s)		2	3.4 ± 1.9	4.3 ± 2.4*

*p < 0.001, two-sample *T*-test or chi-square, as appropriate

**Table 2:** Average and standard deviation of the attenuations per tube voltage group

Tube voltage (kV)	Low attenuation		Regular attenuation	
	*n*	Attenuation (HU)	*n*	Attenuation (HU)
70	111	350 ± 37	357	522 ± 41
80	110	342 ± 35	426	499 ± 37
90	75	348 ± 34	214	506 ± 36
100	39	347 ± 35	29	484 ± 36
110	23	340 ± 27	18	473 ± 33
120	41	312 ± 36	4	431 ± 44

**Fig. 3:**
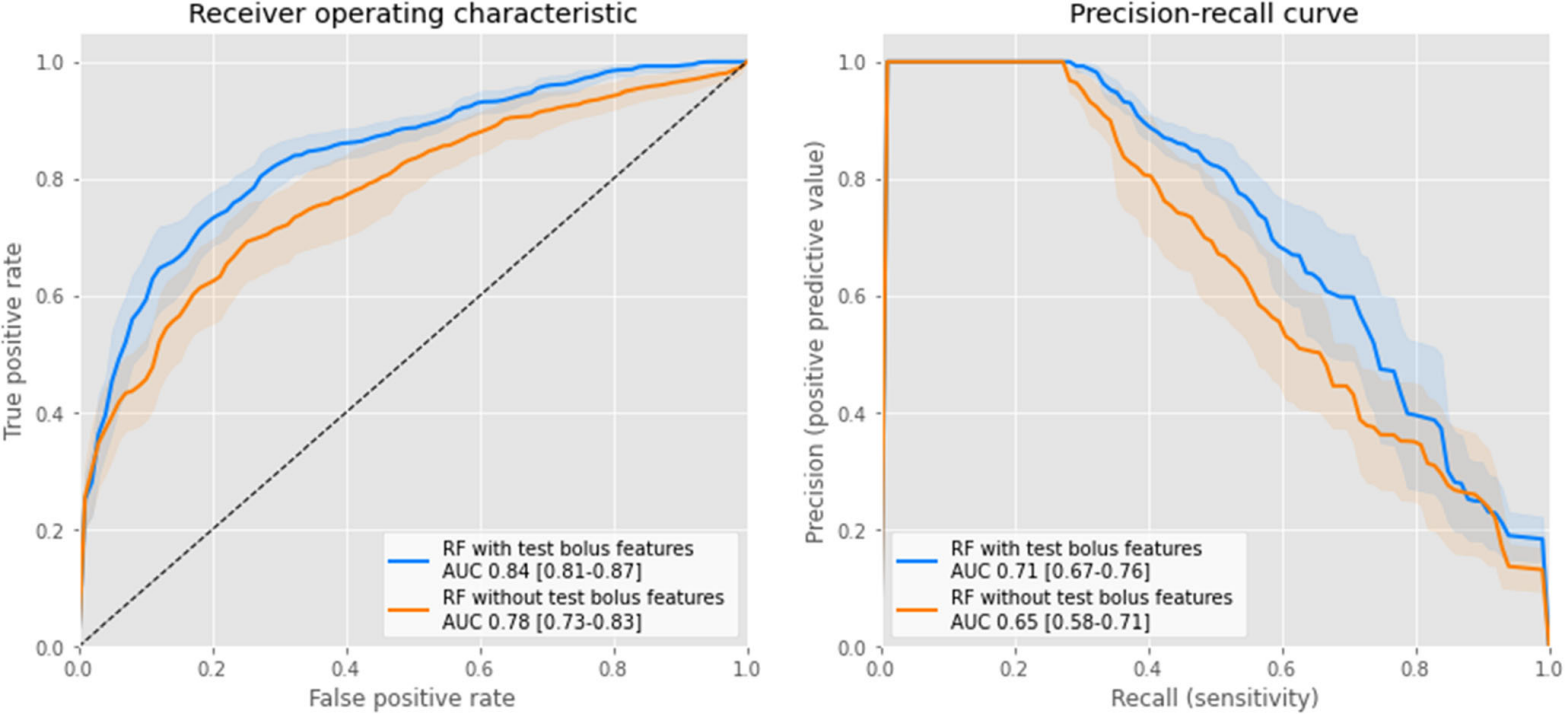
Average receiver operating characteristic (left) and precision-recall curves (right) with 95% confidence interval for models trained with patient features, CT settings, and test bolus features and with only patient features and CT settings. RF = *random forest*, *AUC = area under the curve*

The AUROC and AUPRC together with corresponding 95% CI for all experiments are shown in Table 3. The models with the highest average accuracies were trained with RF and had an AUPRC of 0.71 (95% CI: 0.66–0.76) and AUROC of 0.84 (95% CI: 0.81–0.87), with a sensitivity of 0.66 and specificity of 0.88 (Fig. 3). Notably, these models included the test bolus features. Regarding the models without the test bolus features, the best performing model was also achieved by the RF model. In comparison, this model had an AUROC of 0.78 (95% CI: 0.73–0.83) and AUPRC of 0.65 (95% CI: 0.58–0.71). The differences between the AUROC and AUPRC values for the various models using the test bolus features were not statistically significant. The AUROC difference of the prediction models between using and not using the test bolus features was statistically significant (*p* value = 0.027). The difference in AUPRC between the two models was not statistically significant (*p* value = 0.23).

**Table 3:** Evaluation of the low attenuation detection models with 95% confidence interval. AUPRC = area under the precision-recall curve, AUROC = area under the receiver operating characteristic curve

Model/metric	Including patients features CT settings and test bolus features		Including patients features and CT settings	
	AUPRC	AUROC	AUPRC	AUROC
Logistic regression	0.70 (0.63–0.76)	0.83 (0.79–0.87)	0.62 (0.55–0.68)	0.77 (0.72–0.82)
Random forest	0.71 (0.66–0.76)	0.84 (0.81–0.87)	0.65 (0.58–0.71)	0.78 (0.73–0.83)
XGBoost	0.70 (0.66–0.75)	0.83 (0.80–0.87)	0.64 (0.59–0.69)	0.78 (0.74–0.82)
Support vector machines	0.67 (0.62–0.73)	0.82 (0.78–0.86)	0.56 (0.50–0.63)	0.75 (0.70–0.80)
Neural networks	0.69 (0.63–0.74)	0.82 (0.79–0.86)	0.61 (0.55–0.68)	0.76 (0.71–0.80)

**Fig. 4:**
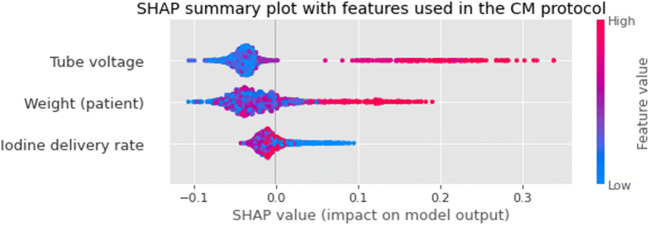
Importance of the CM protocol features (average on the test folds) using SHAP values. The amplitude of the SHAP value indicates the feature importance for the prediction (positive values mean low attenuation). The colors represent the values of the features, with red for high values and blue for low values. *CM* = *Contrast material*

**Fig. 5:**
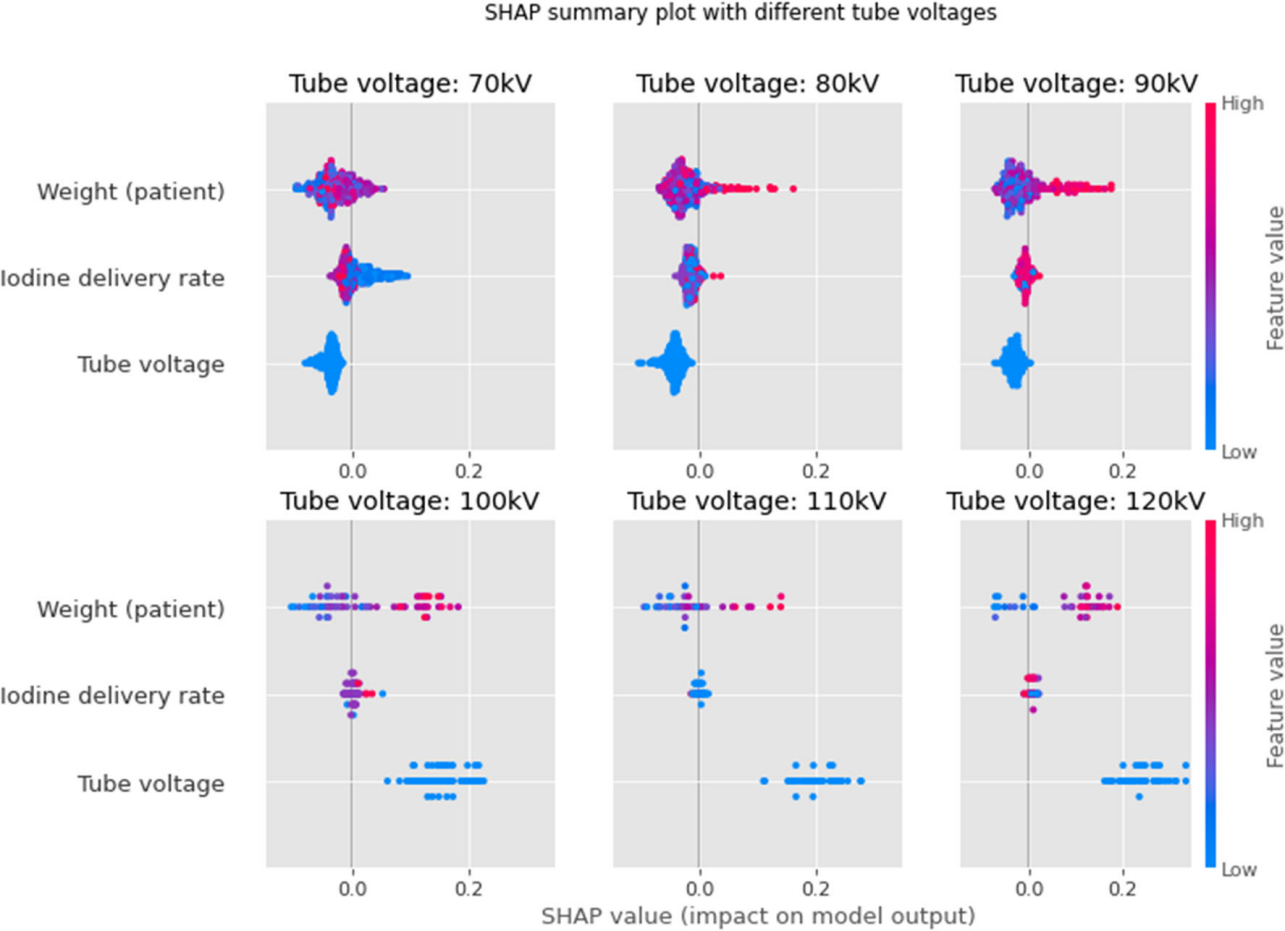
Importance of the CM protocol features (average on the test folds) using SHAP values divided by tube voltage group. The amplitude of the SHAP value indicates the feature importance for the prediction (positive values mean low attenuation). The colors represent the values of the features, with red for high values and blue for low values. Note that there is only one color for the tube voltage since there is only one tube voltage per group. *CM* = *Contrast material*

The SHAP summary plot is presented in Fig. 4, showing only the features related to the CM protocol. As might be expected, it shows that high tube voltages are strongly associated with low attenuations. Furthermore, higher body weights and lower IDR also result in higher chances of insufficient attenuation. As the contrast delivery protocol, used in this study, adjusted the IDR for kV settings and body weight, the effect of these features was evaluated in each tube voltage group (Fig. 5). The kV categories of 70, 80, and 90 kV are associated with intended attenuation values (with a negative SHAP value) and 100, 110, and 120 kV with lower attenuation values (with a positive SHAP value).

**Fig. 6:**
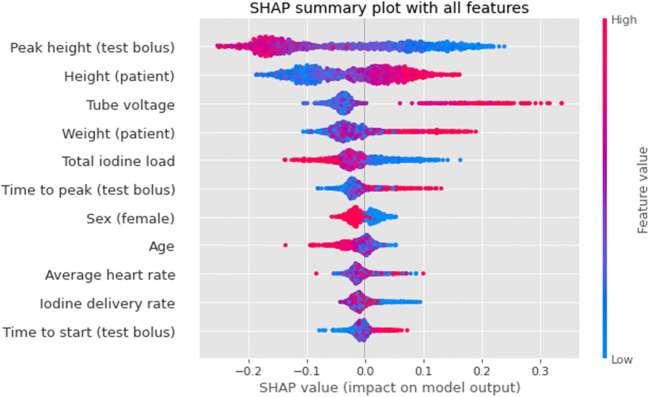
Importance of all features included in the model (average on the test folds) using SHAP values. The amplitude of the SHAP value indicates the feature importance for the prediction (positive values mean low attenuation). The colors represent the values of the features, with red for high values and blue for low values. *CM* = *Contrast material*

Figure 6 shows the SHAP values of all features used in the model. Of all these features, the peak height of the test bolus contrast curve is the most impactful feature (with low peak height associated with low attenuation) followed by body height (high body height values associated with low attenuation). Regarding the protocol features, the tube voltage is the third most important.

## Discussion

In this study, we have shown that ML models are accurate in predicting low attenuation scans. Moreover, in the setting of a patient-specific contrast delivery protocol adjusting the IDR for kV setting and body weight, the peak height of the test bolus curves is the most impacting feature for the model. Including the test bolus features, the prediction accuracy of the models increased, compared to models using only patient features and CT settings. This highlights the association of the test bolus features, specifically the peak height of the test bolus attenuation curve with luminal attenuation and this should be considered when further refining contrast protocols.

In our population, attenuation was inversely associated with kV, despite IDR adjustment for kV settings. These results suggest that it is worth adjusting the IDR even more for kV in our clinical protocol. However, such extra IDR adaptation for kV cannot account for the interplay of other settings on IDR and image quality as indicated by the results showing that multiple parameters influence image quality, most likely in a non-linear fashion. In a previous study, we showed that the patient-tailored contrast delivery protocol contributed to reduced variation in contrast attenuation in the coronary arteries. However, despite the correction for kV and body weight, there remained considerable variation between patients, and coronary attenuation was not sufficiently high in all patients to assure accurate radiologic assessment. Therefore, our study suggests that a straightforward correction for kV settings and body weight underestimates the complexity of the scanning parameters, which do not take the interaction of other parameters with the IDR into account.

Although the peak height of the test bolus was already found significantly associated with image quality in other studies, in this study, we aimed at the application of AI to predict too low coronary artery attenuation in a clinical setting with a contrast protocol adjusted for body weight and kV. The strong association of the test bolus and optimal enhancement in the ascending aorta on the CCTA is not surprising because of its similar signal. However, the test bolus is a small volume of contrast. A longer bolus results in accumulation and therewith a higher plateau of attenuation. The filling time will result in this plateau feature. The form of this upslope and plateau may vary, most likely concerning time to peak and peak value.

### Model performance

We evaluated five different ML techniques and the differences between the accuracy of these models were not statistically significant. All evaluated ML techniques used in this study seem to be able to identify insufficient contrast cases, including LR, which only takes linear relationships between features and outcome into consideration. Using as reference the RF model, with a sensitivity of 0.66 and specificity of 0.88, we can identify 263 (from 399) CTs with a relatively small number of false positives (125). Additionally, the prediction probability threshold could be adjusted to have higher sensitivity at a cost of lowering the specificity.

### Comparison with previous studies

Multiple studies aim to use ML to improve the CT acquisition process and image quality [20]. Also, some studies aimed on developing patient-tailored CM protocols using the test bolus features in 100–120 kV scans, not covering the currently available kV range 70–120 kV [21, 22].

Besides the use of test bolus, some studies use tailored CM protocols with (automatic) bolus tracking. Martin et al [23] evaluated the feasibility of a vendor’s software using a tube voltage-tailored CM application, which still resulted in more than 25% of the CTAs with attenuations in the ascending aorta below 400 HU. In another study with bolus tracking, Yin et al [24] evaluated protocols tailored for BMI or BSA, and, either way, cases of insufficient attenuation in the aorta occurred. The use of AI, as presented in the current study, could potentially improve different protocols by automatically detecting cases with insufficient attenuation when using a test bolus protocol.

### Limitations

This study was performed with a relatively large cohort; however, due to the retrospective nature of the study design, some patient-specific features were incomplete. Furthermore, this is a single-center study and the CTCAs were acquired with a specific protocol, making the ML models not generally suitable for different protocols without re-training them with additional data. Also, the selected cutoff value, 400 HU for the ascending aorta is arbitrary. However, it should be noted that this value is not the only marker of high-quality coronary CTA. Moreover, the quality was addressed by the (objective) attenuation assessment whereas the quality could also have been addressed by the (subjective) radiologist’s rating. However, it should be noted that this value is not the only marker of high-quality coronary CTA. Moreover, the quality was addressed by the (objective) attenuation assessment whereas the quality could also have been addressed by the (subjective) radiologist’s rating.

Regarding the ML techniques used in this study, all models tested achieved similar accuracies. It might be explained by the limited number of features considered for this analysis. The addition of more features, such as information extracted from the test scan or engineered features, would add additional value that could be exploited by the techniques that can handle a large number of features and nonlinearities. Although attenuation is an important topic regarding image quality of CCTAs, it does not cover image quality completely. Noise, artifacts, or qualitative quality assessments were not considered in this study.

The model can accurately predict low attenuation retrospectively in a large population. Therefore, the current study should be conceived as a proof-of-concept study to predict low attenuation. The effectiveness of the proposed prediction model needs to be addressed in a subsequent prospective study. Also, the extent to which the IDR should be adjusted was beyond the scope of this study. Regression models to estimate the attenuation itself, instead of a binary classification, may be a solution. With a correct estimation of the attenuation, the IDR could be adjusted such that the predicted attenuation is close to acquired luminal attenuation that should be close to the desired value. This approach will be explored in a further study.

Although the peak height of the test bolus was already found significantly associated with image quality in other studies [8, 9], in this study, we aimed at the application of AI to predict too low coronary artery attenuation in a clinical setting with a contrast protocol adjusted for body weight and kV. The strong association of the test bolus and optimal enhancement in the ascending aorta on the CCTA is not surprising because of its similar signal. However, the test bolus is a small volume of contrast. A longer bolus results in accumulation and therewith a higher plateau of attenuation. The filling time will result in this plateau feature. The form of this upslope and plateau may vary, most likely concerning time to peak and peak value.

An infrequent but important factor for inadequate contrast media arrival is dynamic venous compression in the thoracic outlet region. Although technicians are trained in patient positioning to avoid venous compression for optimal contrast dynamics, dynamic venous compression can not be ruled out entirely and this might have also contributed to low attenuation in some patients, which is not accounted for in the current analysis.

### Clinical implications

Current fixed CM delivery protocols may be too simple for adequate contrast enhancement in CTCA. An important step in improving patient-tailored contrast delivery protocols is to understand why and when current approaches fail. Predicting when the protocol is potentially failing is the first step to develop more robust protocols. With the models developed in this study, insufficient attenuation can accurately be predicted and adjustments (such as increasing the IDR) can be performed to avoid too low attenuation. This study also shows the potential value of the information that can be extracted from the test bolus which can be incorporated in more advanced and robust protocols.

## Conclusion

We demonstrate that ML is accurate in the prediction of CCTA with insufficient attenuation on our local imaging protocol. We have shown that, in a protocol already adjusting for kV and body weight, the most impacting feature for the ML model is the peak height of the test bolus curve. Our findings support the development of more refined and more robust patient-tailored contrast delivery protocols with the inclusion of test bolus features. Also, it should be noted that the approach is general and could be applied to a wide range of scanning protocols.

## Supplementary information

ESM 1 (DOCX 102 kb)

## Abbreviations

AUPRC: Precision-recall curves
AUROC: Area under the receiver operating characteristic
BSA: Body surface area
CI: Confidence interval
CM: Contrast material
CO: Cardiac output
CT: Computed tomography
CTCA: Computed tomography coronary angiography
CV: Cross-validation
HU: Hounsfield units
IDR: Iodine delivery rate
kV: Kilovolt
LR: Logistic regression
ML: Machine learning
NN: Neural networks
RF: Random forest
SHAP: Shapley Additive exPlanations
SVM: Support vector machines
TIL: Total iodine load
XGB: Extreme gradient boosting

## Declarations

### Guarantor

The scientific guarantor of this publication is RN Planken and HA Marquering.

### Statistics and biometry

No complex statistical methods were necessary for this paper.

### Study subjects or cohorts overlap

Some study subjects or cohorts have been previously reported in doi: 10.1097/rti.0000000000000593. Overlap of 154 patients (1364 patients were used on this study).

### Methodology

• retrospective

• experimental

• performed at one institution
