# Supplementary material for: Machine learning-based prediction of insufficient contrast enhancement in coronary computed tomography angiography
Source: Eur Radiol. 2022 Jun 16;32(10):7136–45. doi: 10.1007/s00330-022-08901-5 (PMC9474338; doi:10.1007/s00330-022-08901-5)
Supplement: Supplementary file 1 — (DOCX 102 kb) [file 330_2022_8901_MOESM1_ESM.docx]

# Supplementary material

## Analysed classifiers

LR is the standard technique for clinical models. LR is a linear model and is not able to handle the nonlinearities in the data properly. The RF and XGB are an ensemble of decision trees, with different approaches, leading to more complex models but these models can deal with the non-linearity. The SVM and NN are traditional and well-established ML techniques and are also able to deal with the non-linearity of the data differently: the SVM uses hyperplanes in a multidimensional space and the NN uses multiple hidden layers with non-linear activation functions.


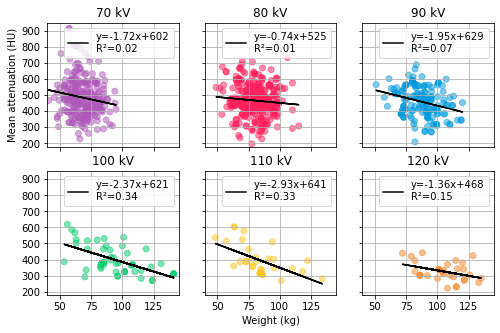
 **Figure I.** Scatter plot showing the relationship between mean attenuation and patient weight per kV group.

**Table I.** Hyperparameters grid used for SVM.

| Classifier | Kernel type | Penalty parameter C | Kernel coefficient γ | Degree of the Polynomial kernel | Class weight |
| --- | --- | --- | --- | --- | --- |
| **SVM** | Linear | [0.1, 1, 10, 100, 1000] | n.a. | n.a. | [None, Balanced] |
|  | Radial basis function | [0.1, 1, 10, 100, 1000] | [1, 0.1, 0.01, 0.001] | n.a. | [None, Balanced] |
|  | Polynomial | [0.1, 1, 10, 100, 1000] | [1, 0.1, 0.01, 0.001] | [2, 3, 4] | [None, Balanced] |
|  | Sigmoid | [0.1, 1, 10, 100, 1000] | [1, 0.1, 0.01, 0.001] | n.a. | [None, Balanced] |

**Table II.** Hyperparameters grid used for RF, GB, and NN.

| Classifier | Parameter name | Parameter value |
| --- | --- | --- |
| **RF** | Number of trees | [50, 100, 200, 500] |
|  | Max features | [auto] |
|  | Max depth | [2, 4, 8] |
|  | Min samples per split | [2, 4, 8] |
|  | Min samples per leaf | [1, 2, 4] |
|  | Class weight | [None, Balanced] |
| **XGB** | Number of trees | [1000] |
|  | Max depth | [3, 6, 12] |
|  | Gamma | [0, 1, 5, 10] |
|  | Subsample | [0.9, 0.7] |
|  | Learning rate | [0.1, 0.05] |
|  | Col sample by tree | [1, 0.7] |
|  | Min child weight | [1, 5] |
|  | Early stopping rounds | 10 |
|  | Eval set size | 0.1 |
|  | Scale pos weight | [1, 2, 3, 4] |
| **NN** | Activation | [relu] |
|  | Hidden layer sizes | [50], [100], [10, 10], [50, 50], [5, 5, 5], [10, 10, 10], [50, 50, 50] |
|  | Alpha | [0.001, 0.0001] |
|  | Solver | [adam] |
|  | Learning rate | [adaptive] |
|  | Initial learning rate | [0.1, 0.01, 0.001] |
|  | Early stopping | True |
|  | N iter no change | 10 |
| **LR** | Penalty | [L1, L2] |
|  | Solver | [Liblinear, lbfgs] |
|  | C | [0.1, 1, 10] |

**Table III.** Average LR coefficients over folds.

| Fold | *1* | *2* | *3* | *4* | *5* | *6* | *7* | *8* | *9* | *10* | *Average* |
| --- | --- | --- | --- | --- | --- | --- | --- | --- | --- | --- | --- |
| Peak height - test bolus (HU) | -0.02 | -0.02 | -0.02 | -0.02 | -0.02 | -0.02 | -0.02 | -0.02 | -0.02 | -0.02 | -0.02 |
| Time to peak - test bolus (s) | 0.17 | 0.17 | 0.16 | 0.14 | 0.15 | 0.14 | 0.13 | 0.17 | 0.20 | 0.14 | 0.16 |
| Time to start - test bolus (s) | -0.14 | -0.10 | -0.10 | -0.05 | -0.11 | -0.09 | -0.07 | -0.10 | -0.13 | -0.09 | -0.10 |
| Age (yrs) | -0.02 | -0.02 | -0.01 | -0.02 | -0.01 | -0.01 | -0.01 | -0.02 | -0.01 | -0.01 | -0.02 |
| Height (cm) | 0.06 | 0.06 | 0.06 | 0.05 | 0.05 | 0.05 | 0.06 | 0.05 | 0.07 | 0.06 | 0.06 |
| Weight (kg) | 0.02 | 0.03 | 0.03 | 0.03 | 0.03 | 0.02 | 0.02 | 0.03 | 0.02 | 0.03 | 0.03 |
| Average heart rate (bpm) | 0.01 | 0.03 | 0.02 | 0.02 | 0.02 | 0.02 | 0.02 | 0.02 | 0.02 | 0.02 | 0.02 |
| Iodine delivery rate (g I/s) | 1.58 | 0.40 | 1.80 | 1.15 | 0.42 | 0.69 | 0.83 | 0.94 | 1.74 | 0.55 | 1.01 |
| Total iodine load (g) | -0.68 | -0.73 | -0.80 | -0.65 | -0.62 | -0.60 | -0.60 | -0.60 | -0.77 | -0.54 | -0.66 |
| Sex (female) | 0.12 | -0.08 | -0.06 | 0.05 | 0.01 | -0.18 | -0.05 | -0.13 | 0.05 | 0.14 | -0.01 |
| Tube voltage 70 kV | -3.81 | -3.19 | -4.31 | -2.48 | -3.86 | -2.52 | -3.79 | -2.40 | -4.45 | -2.40 | -3.32 |
| Tube voltage 80 kV | -3.53 | -2.57 | -3.72 | -2.06 | -3.37 | -2.12 | -3.45 | -2.10 | -3.82 | -2.00 | -2.87 |
| Tube voltage 90 kV | -2.10 | -0.79 | -2.08 | -0.82 | -1.88 | -0.72 | -1.93 | -0.73 | -2.21 | -0.79 | -1.41 |
| Tube voltage 100 kV | 0.00 | 1.45 | 0.00 | 1.24 | 0.22 | 1.31 | 0.27 | 1.12 | 0.07 | 1.07 | 0.68 |
| Tube voltage 110 kV | 0.00 | 1.78 | 0.16 | 1.47 | 0.00 | 1.17 | 0.00 | 1.38 | 0.00 | 1.06 | 0.70 |
| Tube voltage 120 kV | 1.61 | 3.16 | 2.53 | 2.56 | 1.84 | 2.89 | 1.65 | 2.54 | 3.04 | 2.61 | 2.44 |
